# Body Image Dissatisfaction among Adolescent Girls of a Municipality in Kathmandu: A Cross-sectional Survey

**DOI:** 10.31729/jnma.5378

**Published:** 2021-09-30

**Authors:** Garima Malla, Sarala Pradhan Joshi, Alisha Thapa

**Affiliations:** 1 Department of Public Health, Om Health Campus, Kathmandu, Nepal

**Keywords:** *adolescent*, *body dissatisfaction*, *body image*

## Abstract

**Introduction:**

Body image is the person's perceptions, thoughts and feelings about his/her body which is a multi-dimensional concept. Body Image Dissatisfaction can be measured using a Body Shape Questionnaire. The main aim of this study was to find out the prevalence of body image dissatisfaction among adolescent girls of a municipality in Kathmandu.

**Methods:**

A cross-sectional survey was done from July 2019 to December 2019 among the female students from grade eight to twelve of the selected private schools of Budhanilkantha municipality. Stratified random sampling was done. Body Shape Questionnaire short version was used to measure the prevalence of body dissatisfaction. Data were analyzed using Statistical Package for the Social Sciences version 23. Point estimate at 95% Confidence Interval was done, frequency and percentage were calculated.

**Results:**

One hundred ninety seven (75.2%) at 95% Confidence Interval (69.97-80.43) students were found to have body image dissatisfaction, among which Positive Body Image Dissatisfaction was found to be in 85 (42%) respondents which indicates that 112 (58%) respondents with healthy Body Mass Index were dissatisfied with their body shape.

**Conclusions:**

Prevalence of body image dissatisfaction was found to be high in adolescent girls though most of the girls had normal body weight and a healthy Body Mass Index.

## INTRODUCTION

Body image is the person's perception, thoughts and feelings about his/her body.^1^ Adolescents undergo various physical and psychological changes and make adjustments to their appearance.^2^ Nowadays, being thin is desired within societies and adolescents, especially, girls, are dissatisfied with their body and perceive themselves as over/underweight trying to lose/gain weight to achieve the socially endorsed ideal of a beautiful body.^3^

Studies on body dissatisfaction related research have been carried out abundantly in the western countries and few non-western countries. But in Nepal, such researches are yet to be done.^2^ Even though a research has been conducted in Kaski district, there is a greater need of similar researches on the urban areas where adolescents are more exposed to westernization and under the pressure of maintaining ideal thin body.^3^

The main aim of this study was to determine the prevalence of Body Image Dissatisfaction (BID) among adolescent girls of Budhanilkantha Municipality, Kathmandu.

## METHODS

A cross-sectional survey was conducted among 262 female students who were in their middle and late adolescence of age group 14 to 19 from selected schools of Budhanilkantha Municipality after obtaining ethical clearance from the Nepal Health Research Council (Ref no.3363). The survey was done from July 2019 to December 2019. The sample was taken from a total of 2550 students altogether from 5 schools of Budhanilkantha Municipality, Kathmandu. Stratified Random Sampling was done. The data collection period was one month. Parental consent was obtained from participants under 18.

Female participants whose parents did not give consent, who were in their early adolescence and over 19 years of age were excluded. Female participants whose parents gave consent and who were between the age group of 14 to 19 were included in the study. Prevalence i.e. 80.9% was taken from a study conducted in Nepal.

The sample size was conducted using the following formula,

n = Z^2^ × p × q / e^2^

  = 1.96^2^ × (0.809 × 0.191) / 0.05^2^

  = 0.594/0.0025

  = 237.6

  ~238

Where,

n = sample size
p = prevalence, 80.9%^3^
q = 1-p
e = margin of error, 5%
Z = 1.96 at 95% confidence interval

Hence, the sample size calculated was 238. Taking a non-response rate of 10%, the total sample size was 262. The schools were chosen using stratified random sampling and respondents were taken randomly from the schools.

One of the tools used for data collection in the study was a semi-structured questionnaire with the Body Shape Questionnaire short version (BSQ-8C) to assess Body Image Dissatisfaction. It consisted of 8 questions extracted from the full version.^4^ Questions were asked by adding the phrase, "Over the past few weeks".

A psychological tool called the Figure Rating Scale, also known as the Stunkard scale was used to determine bodily dissatisfaction.^5^ It contained nine female schematic silhouettes, ranging from extreme thinness to extreme obesity (representing BMI ranging from 18.2 to 40.6).^6^ The participants were asked to choose the silhouette closest to their body size (perceived) and one that represented their ideal body size (desired). The discrepancy between the two figures was seen as an indication of dissatisfaction. The actual BMI of the participants was calculated by measuring the height and weight of the participants. Pre-testing of the tool was done taking 10% of the total sample size.

Data was entered and analyzed in Statistical Package for the Social Sciences version 23. Prevalence and level of Body Image Dissatisfaction were determined by the total scoring. Questions were scored on a 6-point Likert scale (never, rarely, sometimes, often, very often, always), points ranging from 1 (lowest) for never to 6 for always (highest). The points were then added in such a way that high scores reflected body dissatisfaction and low scores reflected the absence of dissatisfaction.

## RESULTS

Among 262 respondents, the prevalence of Body Image Dissatisfaction was 197 (75.2%) at 95% Confidence Interval (69.97-80.43) (Table 1).

**Table 1:** Prevalence of body image dissatisfaction (n = 262).

Body Image Dissatisfaction	n (%)
Yes	197 (75.2)
No	65 (24.8)

Among respondents with BID 197 (75.2%), Positive Body Image Dissatisfaction was present in 85 (43.1%) participants, which indicated that the remaining 112 (56.9%) participants had Negative Body Image Dissatisfaction i.e., despite having a healthy BMI, they were unsatisfied with their body and how they looked (Table 2).

**Table 2:** Positive and negative body image dissatisfaction (n = 197).

Body Image Dissatisfaction	n (%)
Positive	85 (43.1)
Negative	112 (56.9)

More than half of the respondents i.e.144 (55%) had aslight level of BID. The minority of participants, 21 (8%) were highly dissatisfied with their bodies (Table 3).

**Table 3:** Body dissatisfaction level (n = 197).

Level of Dissatisfaction	n (%)
Slight body image dissatisfaction	144 (55)
Mild body image dissatisfaction	32 (12.2)
High body image dissatisfaction	21 (8)

Perceived BMI levels were the ones that respondents with BID thought their current body shape and BMI was at the time of response, the desired BMI levels were the ones that respondents with BID wanted their body shape and BMI to be like (ideal shape). Four (2%) respondents with BID chose the thinnest figure with underweight BMI as their current shape, meanwhile most of the respondents with BID chose figures with normal BMI. 38 (19.3%) respondents with BID chose figure with underweight BMI as their desired body shape while the rest of the respondents with BID chose figure with normal BMI (Table 4).

**Table 4:** Perceived and desired Body Mass Index (n = 197).

Perceived Body Mass Index (kilogram per meter square)	n (%)
18.2 (Underweight)	4(2.0)
19.3 (Normal weight)	27 (13.7)
20.9 (Normal weight)	53 (26.9)
23.1 (Normal weight)	80 (40.6)
26.2 (Over-weight/Obese)	28 (14.2)
29.9 (Over-weight/Obese)	5(2.6)
Desired Body Mass Index (kilogram per meter square)	n (%)
18.2 (Underweight)	38 (19.3)
20.9 (Normal weight)	110 (55.8)
23.1 (Normal weight)	49 (24.9)

Among 197 respondents with Body Image Dissatisfaction, 172 (87.3%) were in their middle adolescence. Half of the respondent's ethnicity was Brahmin/Chhetri 101 (51.3%), followed by Janajatis 83 (42.1%). More than half of the girls with BID had normal body weight 108 (54.8%) followed by underweightgirls 71 (36.1%). The number of respondents with BID who were below Secondary Education Examination (SEE) level was higher 117 (59.4%) than those who were above SEE level 80 (40.6%). About two-thirds of the respondents were from urban areas 134 (68%) while the rest of the participants with BID were from rural areas 63 (32%). Similarly, close to two-thirds of respondents 129 (65.5%) were moderately exposed to media (Table 5).

**Table 5:** Socio-demographic characteristics (n = 197).

Characteristics	n (%)
**Age**	
14-17	172 (87.3)
18-19	25 (12.7)
**Body Mass Index**	
Underweight	71 (36.1)
Normal weight	108 (54.8)
Overweight	16 (8.1)
Obese	2 (1.0)
**Ethnicity**	
Brahmin/Chhetri	101 (51.3)
Janajati	83 (42.1)
Others	13 (6.6)
**Educational level**	
Below SEE	117 (59.4)
Above SEE	80 (40.6)
**Permanent place of residence**	
Urban	134 (68.0)
Rural	63 (32.0)
**Media exposure**	
Poor	38 (19.3)
Moderate	129 (65.5)
High	30 (15.2)

## DISCUSSION

In the present survey, it was found that almost 80% of the respondents were dissatisfied with their Body Image which is quite similar to the results of the study conducted in Kaski district of Nepal. In the study, four out of five (80.9%) adolescent girls were dissatisfied with their body image where positive body image dissatisfaction was (41.7%) that is almost equal to the findings of this survey. This might be because of the similar target group and a similar sample size.^3^ Misconceptions about own body were found to be high among the participants of both the study.

Further, the present survey showed the percentage of participants who were satisfied with their body weight was around one-fourth of the total participants, i.e., 24.8% which is not far from the findings of the study conducted in Taibah University in 2011 that showed about 26.4% participants with Body Image Satisfaction.^1^ Women tend to be critical of their appearance and place importance on the way they look.

In the same way, a study was conducted in 5 boarding schools of Ringroad area, Kathmandu, Nepal among 100 adolescents with the objective to find out the perception of Nepalese adolescents on their own and other's body image and to identify the effects of body image and body dissatisfaction on adolescents. The study showed that about 80% of adolescent girls wanted to change something about their overall appearance indicating dissatisfaction with their current body shape which is comparably near to the BID prevalence of the current survey (BID=75.2%).^2^

The present survey showed that the respondents with normal body weight were more dissatisfied with their body weight (56.9%) which is in contrast with the findings of the study among Brazilian adolescents that included 71,740 students. The study showed that more students who were underweight or overweight/obese had higher BID. This might be because of the bigger sample size and different target group (early adolescents) of the nationwide study compared with our smaller sample size and target group of middle and late adolescent girls only.^7^

Furthermore, in a study performed in Iran, among all participants, the underweight (41.66%), normal weight (67.71%) and overweight (57.14%) categories of BMI selected the thinnest figure as their desirable or ideal body image perception. But in our study, only about twenty percent (19.3%) of all the respondents chose the thinnest figure as their desired body shape. This might be because of the complete difference in the target population, i.e. in the study; it was 18-35 wherein the present study it is 14-19.^8^

A study conducted in the United States concluded that body dissatisfaction and weight and shape concern intensify across adolescence, but associations between the constructs and BMI remain gender-specific. Similarly, due to the present survey being based on adolescents, it had a high prevalence of Body Image Dissatisfaction among adolescent girls. Also, this s was done specifically among female students that is why high dissatisfaction percent might have occurred.^9^

In the end, a cross-sectional study similar to our current survey between 8,925 US residents and 742 non-US residents between the ages of 18 to 40 was performed to examine the association between body mass index and well-being. Measures of weight-based body dissatisfaction, body appreciation, and subjective happiness, and demographic data including their education, age, and body mass index (BMI) was provided by the respondents. The study showed only around 10% of the respondents being satisfied with their Body Shape referring to about 90% BID which is close yet comparatively larger than the BID findings of this research paper (P=75.2%).^10^

Our study has some limitations. Firstly, BSQ-8C is not the full version of the questionnaire, so it does not cover all the aspects of body image. Also, convenient sampling was done to select the study area which is limited to only a municipality in Kathmandu. Therefore, it cannot be generalized concerning whole country settings.

## CONCLUSIONS

From this study, we can see that more than half of the girls even with a healthy BMI were not content with their body shape. These results may have implications for raising awareness on body image and increasing dissatisfaction with weight status at family, community and organizational level. Larger studies including participants from all genders should be conducted to determine the prevalence of BID and to prevent it among adolescents at the regional and national levels.
